# Lifelong Craniofacial Growth: Clinical Implications for Osseointegrated Implants

**DOI:** 10.7759/cureus.92703

**Published:** 2025-09-19

**Authors:** Akshim Rana, Shubham K Srivastava, Chinmoy Sikdar, Shitij Srivastava, Abhinav Shekhar

**Affiliations:** 1 Department of Prosthodontics, Sardar Patel Post Graduate Institute of Dental and Medical Sciences, Lucknow, IND

**Keywords:** craniofacial growth, dental implants, implant supported prosthesis, osseointegration, prosthodontics

## Abstract

Endosseous dental implants have demonstrated high clinical success rates, with outcomes consistently replicated across diverse clinical settings. Over time, definitions of implant success have evolved from basic parameters such as pain-free function and absence of infection to more comprehensive criteria, including peri-implant bone stability, marginal bone loss thresholds, intimate bone-to-implant adaptation, and soft-tissue harmony essential for optimal esthetics. While complete-arch, implant-supported rehabilitations within edentulous jaws may maintain a static relationship over time, mixed dentition scenarios involving both natural teeth and implants are influenced by the dynamic nature of craniofacial growth. Although osseointegration and peri-implant tissue stability may persist for decades, the adjacent dentition and jaw structures continue to undergo subtle lifelong positional changes. This disparity can result in functional or esthetic discrepancies despite meeting all conventional success benchmarks. Consequently, the criteria for long-term implant success must consider the impact of lifelong craniofacial growth, which may gradually compromise the harmony between static implant restorations and the continually adapting natural dentition.

## Editorial

Dental implants may remain osseointegrated for decades, but the supporting craniofacial structures are never static. Continuous skeletal remodeling and dental eruption can gradually alter occlusion and esthetics, underscoring the need for long-term monitoring. Osseointegrated dental implants are a highly predictable and durable solution for restoring partial edentulism, with survival rates and functional outcomes supported by decades of clinical use [1]. Success definitions have evolved from focusing solely on implant survival and absence of pathology to incorporating bone stability, minimal bone loss, intimate osseointegration, and harmonious peri-implant soft tissue [1]. These criteria, however, assume that once osseointegration is achieved in a fully mature patient, the implant-jaw relationship remains static. Long-term evidence challenges this assumption, showing that craniofacial growth, although subtle, persists throughout adulthood and can influence the functional and esthetic integration of implants with adjacent dentition [1,2].

We present a common scenario that illustrates this point: a patient in her early 30s receives a single maxillary central incisor implant. The result is flawless for a decade. Then, during a routine review, the implant crown appears slightly shorter than the adjacent tooth, and the gingival margin seems apically displaced. The implant is perfectly stable, yet lifelong growth has shifted the relative position of the surrounding dentition and soft tissue, compromising the esthetics.

Growth in the craniofacial skeleton is not confined to childhood or adolescence. While rapid developmental changes occur in early life, longitudinal studies demonstrate continued eruption of teeth, increases in anterior facial height, and positional changes in the dentoalveolar complex well into adulthood [3,4]. These changes are driven by slow but continuous bone remodeling, periodontal ligament-mediated eruption, and adaptive responses of the dentition to occlusal and functional demands. Facial growth type may also influence patterns of posterior mandibular rotation in females versus anterior rotation in males and should be part of preoperative evaluation [4,5]. Animal experiments have shown that implants remain ankylosed and static relative to surrounding bone during jaw growth, sometimes impeding localized alveolar development [6]. Similar observations in humans confirm that implants placed before or near skeletal maturity can become infraoccluded over time, and that even in adults, natural teeth continue to migrate while implants remain fixed [2,5,7].

When implants are placed alongside natural teeth, their inherent biological differences may lead to three major long-term issues. The first is related to occlusion, as natural teeth in the implant-supported or opposing arch may continue to erupt or drift facially, while the implant remains fixed in position [8]. Second, loss of interproximal contact - most often mesial to the implant crown - can occur due to mesial drift of natural teeth, with reported incidences reaching up to 40% over time [8]. Third, anterior esthetic discrepancies develop when natural incisors extrude and tip lingually, altering incisal edge levels, gingival margin heights, and facial contour alignment relative to the implant restoration [7,9]. Thinning of the labial soft tissues over implants can further accentuate these discrepancies, sometimes leaving no predictable corrective option short of restoration replacement.

These phenomena demand a reconsideration of what constitutes long-term success in implant dentistry. An implant restoration may fulfill current stability and health criteria while still being compromised by changes in the surrounding dentoalveolar framework [9]. Expanded success definitions should address the stability of tooth-implant relationships over time, particularly in cases involving mixed dentitions.

Clinically, this awareness should influence patient selection, timing of placement, and prosthetic design. Chronological age alone is insufficient; skeletal maturity, facial growth pattern, and dental maturation should be assessed before placement [10]. In borderline cases, delaying implant therapy or using interim removable or resin-bonded restorations may be prudent until growth stabilizes. For definitive prostheses, avoiding a rigid connection between implants and natural teeth allows for independent adaptation. Screw-retained designs are preferable to cement-retained designs in high-risk cases, as they facilitate retrieval and modification. In the esthetic zone, choosing restorative contours that permit minor reshaping can help maintain harmony if adjacent teeth erupt over time. Maintenance protocols should emphasize regular clinical and radiographic monitoring, with occlusal adjustments, contact point re-establishment, or prosthetic modification as indicated [10].

Future research should aim to develop predictive tools for identifying patients at higher risk of clinically significant adult craniofacial growth. Standardized classification systems for recording growth-related changes in implant patients would facilitate both research and clinical decision-making. Investigating the growth potential of edentulous areas without implants may also guide optimal timing for intervention [11,12]. Lifelong craniofacial growth, though often subtle, has undeniable clinical implications for implant therapy. Even in fully grown adults, continuous skeletal remodeling, dentoalveolar changes, and soft tissue adaptations can lead to progressive discrepancies between implants and adjacent teeth or facial structures. Such changes may manifest as midline shifts, infraocclusion, or altered esthetics over decades, particularly in the anterior maxilla, where the demand for long-term functional and esthetic stability is highest [2,7,9].

For clinicians, the key takeaway is clear: implant placement must extend beyond the immediate restorative outcome and anticipate the patient’s lifelong craniofacial growth trajectory. This requires careful case selection, meticulous three-dimensional planning, and a preference for prosthetic designs that allow for retrievability and future modification [10]. Patients should be counseled about the possibility of long-term changes, and follow-up protocols should include periodic esthetic and occlusal reassessment [9,10]. Ultimately, recognizing and respecting lifelong craniofacial growth is essential for ensuring the enduring success of implant-supported rehabilitations [1,11].
